# Supplementary material for: Providing Scale to a Known Taxonomic Unknown—At Least a 70-Fold Increase in Species Diversity in a Cosmopolitan Nominal Taxon of Lichen-Forming Fungi
Source: J Fungi (Basel). 2022 May 8;8(5):490. doi: 10.3390/jof8050490 (PMC9146994; doi:10.3390/jof8050490)
Supplement: Supplementary file 1 [file jof-08-00490-s001.zip › S4_bPTP_results.pdf]

Max likelihood partition

Species 1 (support = 0.315) = ASAP ‘polytropa41’

polytropa41__111_LLJ20_28; polytropa41__P266_19_66369; polytropa41__P168_19_66369; polytropa41__107_LLJ20_22

Species 2 (support = 1.000) = ASAP ‘chlorophaeodes2’

chlorophaeodes2__AF159927

Species 3 (support = 0.350) = ASAP ‘polytropa61’

polytropa61__P240_ZYY_39; polytropa61__sl21298; polytropa61__P166_19_66372; polytropa61__266_20_66578; polytropa61__P239_ZYY_38

Species 4 (support = 0.093) = ASAP ‘somervellii’

somervellii__16_52913; somervellii__18_60881; somervellii__P286_20_69190; somervellii__18_61201; somervellii__16_53092; somervellii__16_54770; somervellii__16_52899; somervellii__16_53086; somervellii__1_9_19_65622; somervellii__16_53579; somervellii__16_54069; somervellii__16_53507

Species 5 (support = 0.323) = ASAP ‘polytropa22’

polytropa22__MK970662; polytropa22__JX036076; polytropa22__JX036037

Species 6 (support = 0.848) = ASAP ‘polytropa16’

polytropa16__tm14551; polytropa16__tm22539

Species 7 (support = 0.062) = ASAP ‘polytropa42’ & ‘polytropa43’

polytropa42__6842; polytropa42__KU934869; polytropa43__79_16_52934; polytropa43__S79_16_52934

Species 8 (support = 0.477) = ASAP ‘polytropa40’

polytropa40__sl18598q; polytropa40__sl18598v1GENOME; polytropa40__sl18598z; polytropa40__sl18598v1; polytropa40__262_20_66573

Species 9 (support = 0.302) = ASAP ‘concolor1’ & ‘concolor2’

concolor1__AF070037; concolor2__HQ650678

Species 10 (support = 1.000) = ASAP ‘polytropa63’

polytropa63__tm17695

Species 11 (support = 1.000) = ASAP ‘polytropa62’

polytropa62__DQ534470

Species 12 (support = 0.501) = ASAP ‘chlorophaeodes1’

chlorophaeodes1__AF070029; chlorophaeodes1__AY398704

Species 13 (support = 1.000) = ASAP ‘polytropa64’

polytropa64__142_20_67286

Species 14 (support = 1.000) = ASAP ‘intricata03’

intricata_Flakus_29565b

Species 15 (support = 0.104) = ASAP ‘intricata01’ & ‘intricata02’

intricata01__P222_ZYY_135; intricata01__tm13834a; intricata01__tm17204b; intricata01__AF070022; intricata01__AY398703; intricata01__KY266975; intricata02__S272_15_49218; intricata02__tm3614b; intricata02__KY266891

Species 16 (support = 1.000) = ASAP ‘polytropa65’

polytropa65__P223_ZYY_134

Species 17 (support = 0.713) = ASAP ‘polytropa25’

polytropa25__117_LLJ20_37; polytropa25__P229_ZYY_115

Species 18 (support = 0.722) = ASAP ‘polytropa50’

polytropa50__199_20_67021; polytropa50__271_20_66614

Species 19 (support = 0.063) = ASAP ‘solarisA’ & ‘solarisB’

solarisA__MH512981; solarisA__MW257120; solarisA__MW257119; solarisA__MW257121; solarisA__MH512983; solarisA__MH512984; solarisA__MH512982; solarisB__P270_19_64914

Species 20 (support = 0.007) = ASAP ‘polytropa01’ & ‘polytropa02’

polytropa02__KP314321; polytropa02__KP314323; polytropa02__KP314338; polytropa02__KP314376; polytropa02__KP314461; polytropa02__KP314322; polytropa02__tm17811; polytropa02__LICHN603_20; polytropa02__tm17629; polytropa02__tm10003202; polytropa01__sl21283; intricata_Flakus_29519; polytropa01__sl21286; intricata_RodriguezFlakus_3751

Species 21 (support = 1.000) = ASAP ‘polytropa03’

polytropa03__JN873881

Species 22 (support = 1.000) = ASAP ‘polytropa05’

polytropa05__11_20_67481

Species 23 (support = 0.001) = ASAP ‘fuscobrunnea’, ‘polytropa04’ & ‘polytropa25’

fuscobrunnea__JN873875; fuscobrunnea__MK208774; fuscobrunnea__MK208720; fuscobrunnea__MK208760; fuscobrunnea__MK208809; fuscobrunnea__MK208803; fuscobrunnea__JN873877; fuscobrunnea__MK208784; fuscobrunnea__MK208807; fuscobrunnea__MK208817; fuscobrunnea__MK208773; fuscobrunnea__MK208752; fuscobrunnea__MK208799; fuscobrunnea__MK208732; fuscobrunnea__MK208810; fuscobrunnea__MK208804; fuscobrunnea__JN873873; fuscobrunnea__JN873874; fuscobrunnea__MK208806; fuscobrunnea__JN873876; fuscobrunnea__GU170839; fuscobrunnea__GU170840; fuscobrunnea__DQ534484; fuscobrunnea__MK970660; fuscobrunnea__MK208710; fuscobrunnea__MK208816; fuscobrunnea__MK208726; fuscobrunnea__MK208714; fuscobrunnea__JN873872; fuscobrunnea__MK208724; fuscobrunnea__MK208765; fuscobrunnea__MK970661; fuscobrunnea__JX036118; fuscobrunnea__sl20115GENOME; polytropa01__sl20101GENOME;

fuscobrunnea__HQ650643; polytropa01__sl20098GENOME;
polytropa04__71_20_67247; polytropa04__204_20_67090

Species 24 (support = 0.081) = ASAP 'subintricata1' & 'subintricata5'

subintricata1__MT938943; subintricata1__MT938938; subintricata1__MT938939; subintricata1__MT938951; subintricata1__MT938935; subintricata1__MT938952; subintricata1__MT938923; subintricata1__MT938969; subintricata1__MT938985; subintricata1__MT938934; subintricata1__MT938928; subintricata1__MT938940; subintricata1__MT938986; subintricata1__GU480112; subintricata1__MT938946; subintricata5__MT939010

Species 25 (support = 1.000) = ASAP 'subintricata4'

subintricata4__MT939009

Species 26 (support = 0.039) = ASAP 'polytropa51'

polytropa51__215_20_66971; polytropa51__227_20_66787; polytropa51__15_20_69102; polytropa51__246_20_67710; polytropa51__298_20_66724; polytropa51__P250_19_64312; polytropa51__3_20_67471; polytropa51__32_20_67444; polytropa51__251_20_67583; polytropa51__254_20_67584; polytropa51__14_20_69106; polytropa51__41_20_69021; polytropa51__S72_16_50647; polytropa51__5_20_67464; polytropa51__72_16_50647; polytropa51__17_20_69097; polytropa51__33_20_67446

Species 27 (support = 0.891) = ASAP 'polytropa53'

polytropa53__1_69_19_65636; polytropa53__P216_19_65636; polytropa53__1_3_19_63691; polytropa53__P185_19_65279

Species 28 (support = 1.000) = ASAP 'polytropa52'

polytropa52__G28_16_54064

Species 29 (support = 0.505) = ASAP 'polytropa54'

polytropa54__sl18541z; polytropa54__sl18541zGENOME

Species 30 (support = 0.999) = ASAP 'polytropa55'

polytropa55__118_LLJ20_38

Species 31 (support = 0.996) = ASAP 'polytropa66'

polytropa66__268_20_66611

Species 32 (support = 0.996) = ASAP 'polytropa67'

polytropa67__267_20_66584

Species 33 (support = 0.998) = ASAP 'intricata08'

intricata_RodriguezFlakus_3757

Species 34 (support = 0.998) = ASAP 'polytropa06'

polytropa06__P230_ZYY_103

Species 35 (support = 1.000) = ASAP ‘polytropa60’

polytropa60__P267_19_66369a

Species 36 (support = 0.282) = ASAP ‘polytropa44’

polytropa44__6834; polytropa44__6875; polytropa44__6868; polytropa44__6867

Species 37 (support = 0.544) = ASAP ‘polytropa45’

polytropa45__245_20_67689; polytropa45__297_20_66712; polytropa45__248_20_67696

Species 38 (support = 0.987) = ASAP ‘polytropa48’

polytropa48__94_LLJ20_3

Species 39 (support = 0.987) = ASAP ‘polytropa47’

polytropa47__125_LLJ20_51

Species 40 (support = 1.000) = ASAP ‘polytropa49’

polytropa49__122_LLJ20_47

Species 41 (support = 0.339) = ASAP ‘polytropa31’

polytropa31__sl18522a; polytropa31__sl18522b; polytropa31__sl18522aGENOME

Species 42 (support = 1.000) = ASAP ‘polytropa35’

polytropa35__93_LLJ20_2

Species 43 (support = 1.000) = ASAP ‘polytropa39’

polytropa39__121_LLJ20_46

Species 44 (support = 0.341) = ASAP ‘polytropa38’

polytropa38__L0012a; polytropa38__sl16512aGENOME; polytropa38__L0012c; polytropa38__sl16512bGENOME; polytropa38__L0012b; polytropa38__sl21285; polytropa38__sl20113; polytropa38__sl18474aGENOME; polytropa38__sl18474a

Species 45 (support = 0.447) = ASAP ‘polytropa59’

polytropa59__P167_19_66369b; polytropa59__P268_19_66369b; polytropa59__P242_ZYY_34; polytropa59__P241_ZYY_35

Species 46 (support = 0.989) = ASAP ‘polytropa57’

polytropa57__114_LLJ20_33

Species 47 (support = 0.398) = ASAP ‘polytropa23’

polytropa23__tm13834b; polytropa23__tm17204a

Species 48 (support = 0.812) = ASAP ‘polytropa24’ (part 1)

polytropa24__KP314458

Species 49 (support = 0.848) = ASAP ‘polytropa24’ (part 2)

polytropa24__sl18259b

Species 50 (support = 0.880) = ASAP ‘polytropa24’ (part 3)

polytropa24__126_LLJ20_52

Species 51 (support = 0.565) = ASAP ‘dispersoareolata’

dispersoareolata__92_LLJ20_1; dispersoareolata__AF070016

Species 52 (support = 0.979) = ASAP ‘polytropa46’

polytropa46__P237_ZYY_52

Species 53 (support = 0.016) = ASAP ‘polytropa07’

polytropa07__sl18259GENOME; polytropa07__sl21297; polytropa07__sl21296; polytropa07__sl18404b; polytropa07__sl18402; polytropa07__sl18402GENOME; polytropa07__sl18304bGENOME; polytropa07__sl18304b; polytropa07__sl21322; polytropa07__sl21321; polytropa07__sl18259bGENOME; polytropa07__sl21282a; sl21282; polytropa07__sl21282b; polytropa07__sl18370aGENOME; polytropa07__sl18370a; polytropa07__sl18346d; polytropa07__sl20114; polytropa07__sl21295; polytropa07__sl18346dGENOME; polytropa07__sl18356poly; polytropa07__sl18358GENOME; polytropa07__sl18358a

Species 54 (support = 0.997) = ASAP ‘polytropa08’

polytropa08__AF070017

Species 55 (support = 0.494) = ASAP ‘polytropa09’

polytropa09__tm22536; polytropa09__tm2379

Species 56 (support = 0.736) = ASAP ‘polytropa27’

polytropa27__sl18455b; polytropa27__sl18455bGENOME; polytropa27__sl21280

Species 57 (support = 0.414) = ASAP ‘polytropa26’

polytropa26__sl18665poly; polytropa26__sl18669; polytropa26__sl18669GENOME

Species 58 (support = 0.114) = ASAP ‘polytropa28’ & ‘polytropa29’

polytropa28__KP314319; polytropa28__tm21121; polytropa28__LICHN301_19; polytropa29__KP314294; polytropa29__KP314299

Species 59 (support = 0.991) = ASAP ‘polytropa30’

polytropa30__sl21294

Species 60 (support = 1.000) = ASAP ‘polytropa21’

polytropa21__116_LLJ20_35

Species 61 (support = 0.000) = ASAP ‘polytropa10’, ‘polytropa11’, ‘polytropa12’, ‘polytropa13’, ‘polytropa14’, ‘polytropa15’ & ‘polytropa48’

polytropa10b__sl18251a; polytropa10b__sl18282b; polytropa10b__sl18256GENOME; polytropa10b__sl18273b; polytropa10b__sl18282polyGENOME; polytropa10b__sl18376b; polytropa10b__sl18273poly; polytropa10b__sl18405c; polytropa10b__sl18282bGENOME; polytropa10b__sl18583poly;

polytropa10b__sl18251aGENOME; polytropa10a__252_20_67583; polytropa10a__255_20_67573; polytropa10b__sl21299; polytropa10b__sl21301; polytropa10b__sl18455c; polytropa10b__sl18455cGENOME; polytropa10b__sl18256; polytropa10b__sl18521poly; polytropa10b__sl18355poly; sl21135leca; polytropa10b__sl18280c; sl21370a_polytropa; polytropa10b__sl21281; polytropa10b__sl21320; polytropa10b__sl21302; polytropa10b__jh22700; polytropa10b__LICHN777_20; polytropa10b__tm20502; polytropa10b__sl18375b; polytropa10b__sl18375bGENOME; sl21367_polytropaAGG1; polytropa11__272_20_66615; polytropa11__sl21284; sl21337_polytropa; sl21387_polytropa; sl21391_polytropa; sl21353_polytropa; polytropa14__sl20104; polytropa14__sl20104GENOME; polytropa13__L0039; polytropa13__sl16539GENOME; polytropa13__sl20112GENOME; polytropa13__L0050; polytropa13__sl16550GENOME; polytropa12a__264_20_66574; polytropa12a__KP314358; polytropa12a__265_20_66576; polytropa12a__sl17714; polytropa12a__sl17714GENOME; polytropa12b__sl21287a; polytropa12a__P228_ZYY_121; polytropa15__sl20102; polytropa15__sl20103; polytropa15__sl20102GENOME; polytropa15__sl18595; polytropa15__sl18595GENOME

Species 62 (support = 1.000) = ASAP ‘polytropa20’

polytropa20__95_LLJ20_7

Species 63 (support = 0.056) = ASAP ‘polytropa17’ & ‘polytropa18’

polytropa17__137_20_67152; polytropa17__174_20_68348; polytropa17__202_20_68015; polytropa17__20_67942_185; polytropa17__20_67921_179; polytropa17__242_20_67765; polytropa17__177_20_68364; polytropa17__232_20_66815; polytropa17__34_20_67350; polytropa17__235_20_66904; polytropa17__16_53254; polytropa17__186_20_67049; polytropa17__149_20_67166; polytropa17__293_20_66752; polytropa17__148_20_67165; polytropa17__138_20_67153; polytropa17__155_20_67340; polytropa17__6_20_67463; polytropa17__20_67923_163; polytropa17__225_20_66832; polytropa17__133_20_67110; polytropa17__85_20_67226; polytropa17__218_20_67945; polytropa17__87_20_67214; polytropa17__26_20_67431; polytropa17__230_20_66806; polytropa18__R77_16_53294

Species 64 (support = 0.996) = ASAP ‘polytropa19’

polytropa19__1_106_19_65688

Species 65 (support = 0.823) = ASAP ‘polytropa72’

polytropa_Flakus_29565a; polytropa_Flakus_29524

Species 66 (support = 0.961) = ASAP ‘polytropa58’

polytropa58__115_LLJ20_34

Species 67 (support = 0.156) = ASAP ‘polytropa56’

polytropa56__sl18454; polytropa56__sl18454aGENOME; polytropa56__sl18454a; polytropa56__jh23011poly; polytropa56__tm22538; polytropa56__tm32231; polytropa56__tm22537

Species 68 (support = 0.845) = ASAP ‘subintricata2’

subintricata2__MT939007

Species 69 (support = 0.845) = ASAP ‘subintricata3’

subintricata3__MT939008

Species 70 (support = 0.535) = ASAP ‘polytropa34’

polytropa34__tm21094; polytropa34__tm8820; polytropa34__tm2146; polytropa34__tm13266

Species 71 (support = 0.339) = ASAP ‘polytropa23’, ‘polytropa32’ & ‘polytropa33’

polytropa32a__sl18457poly; polytropa32b__sl21310; polytropa33__L0115; polytropa33__sl16615GENOME

Species 72 (support = 0.246) = ASAP ‘polytropa36’

polytropa36__sl18641fGENOME; polytropa36__sl18653GENOME; polytropa36__sl18653; polytropa36__sl18641f; polytropa36__sl18598GENOME; polytropa36__sl18598poly; polytropa36__sl18539a; polytropa36__sl18539aGENOME

Species 73 (support = 0.972) = ASAP ‘polytropa37’

polytropa37__P238_ZYY_41

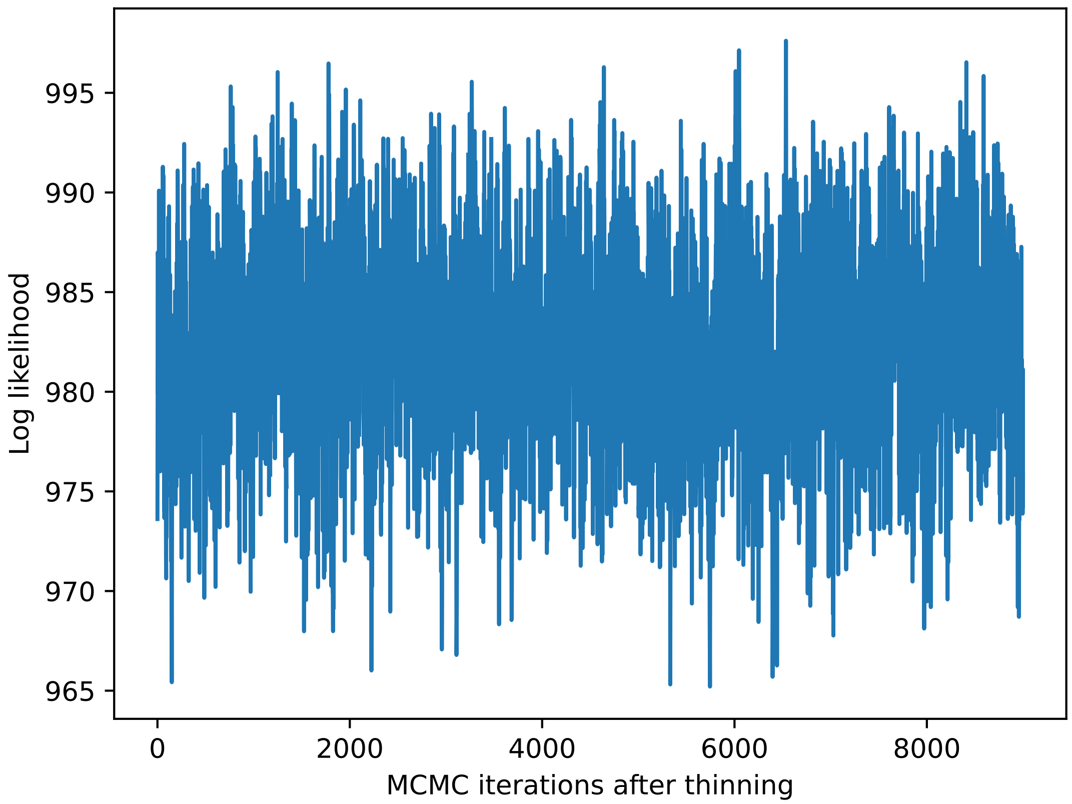
Log likelihood
995
990
985
980
975
970
965
0
2000
4000
6000
8000
MCMC iterations after thinning
