# Supplementary figures and images for: Providing Scale to a Known Taxonomic Unknown—At Least a 70-Fold Increase in Species Diversity in a Cosmopolitan Nominal Taxon of Lichen-Forming Fungi
Source: J Fungi (Basel). 2022 May 8;8(5):490. doi: 10.3390/jof8050490 (PMC9146994; doi:10.3390/jof8050490)

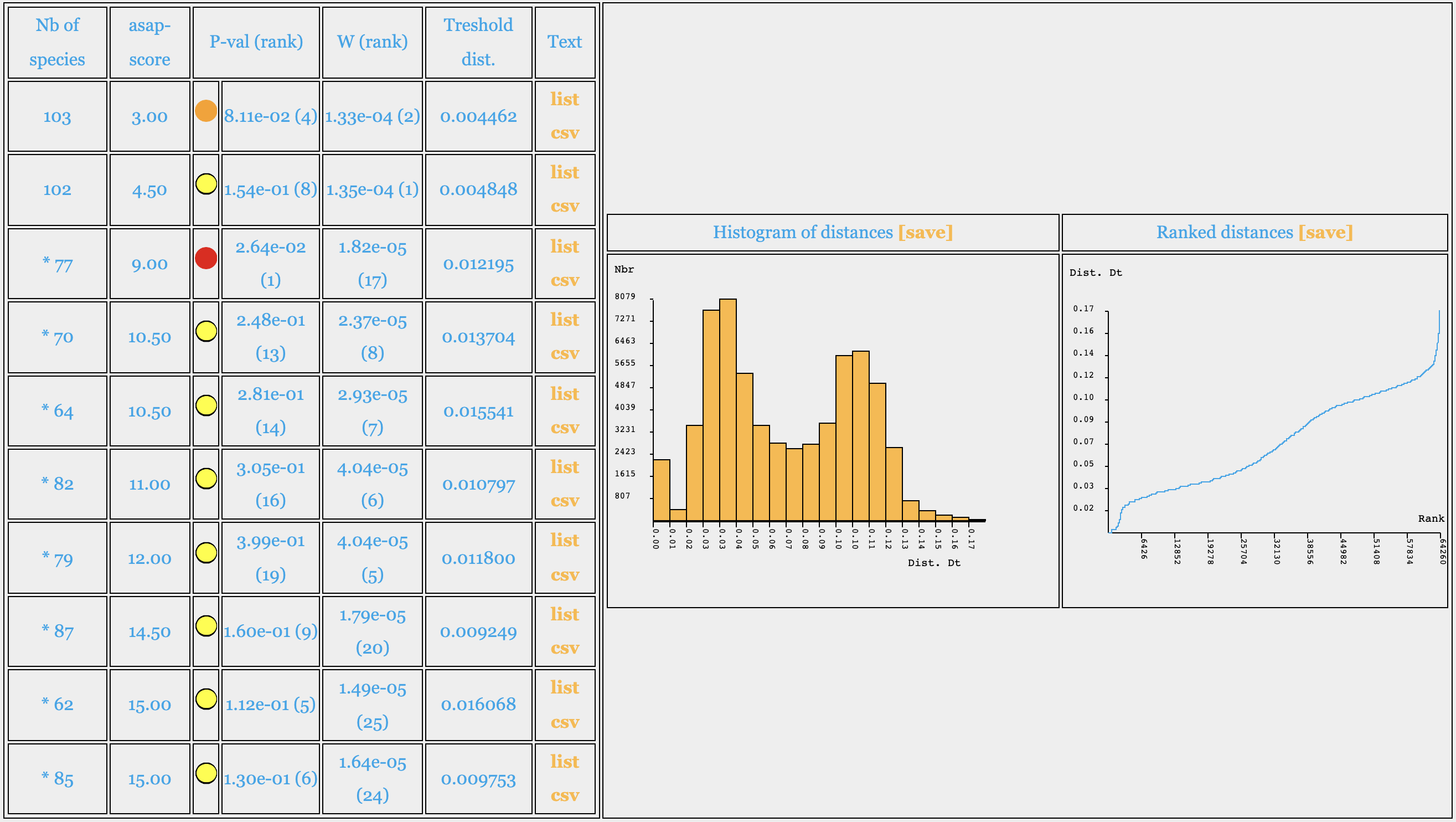

Supplement: Supplementary file 1 [file jof-08-00490-s001.zip › S2_ASAP_rankings_scores_v27jan2022.png]

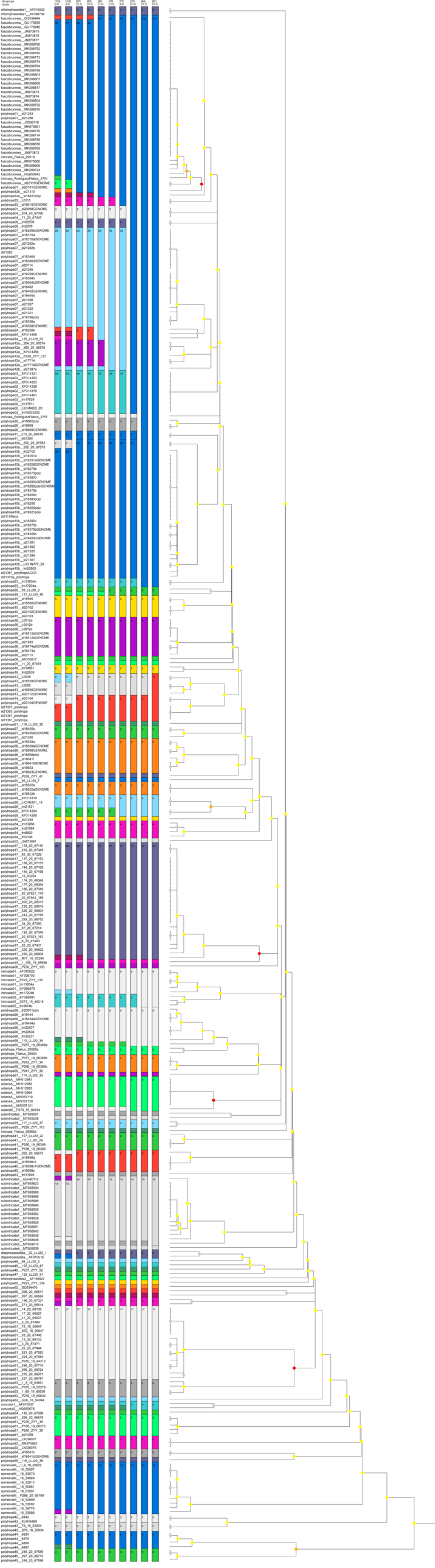

Supplement: Supplementary file 1 [file jof-08-00490-s001.zip › S3_ASAP_tree_w_partitions_v27jan2022.pdf]

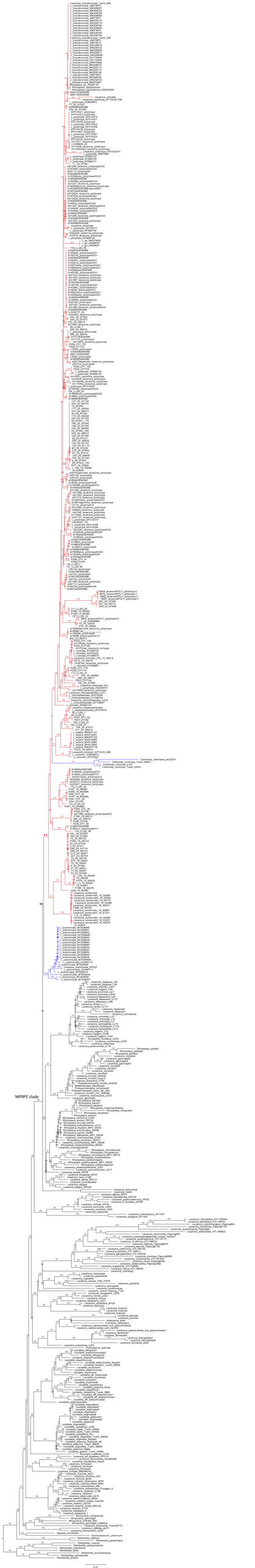

Supplement: Supplementary file 1 [file jof-08-00490-s001.zip › S5_ITS_mtSSU_polytropa_group_monophylyR1.pdf]

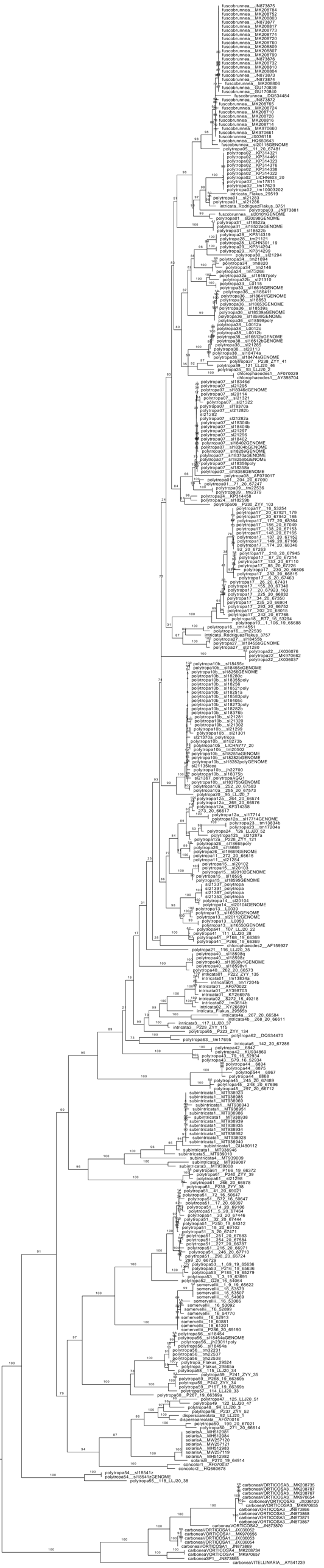

Supplement: Supplementary file 1 [file jof-08-00490-s001.zip › S7_five_gene_topology_27jan2022.pdf]
